# Ablation of sensory nerves favours melanoma progression

**DOI:** 10.1111/jcmm.15381

**Published:** 2020-07-20

**Authors:** Pedro H. D. M. Prazeres, Caroline Leonel, Walison N. Silva, Beatriz G. S. Rocha, Gabryella S. P. Santos, Alinne C. Costa, Caroline C. Picoli, Isadora F. G. Sena, William A. Gonçalves, Mariana S. Vieira, Pedro A. C. Costa, Leda M. C. C. Campos, Miriam T. P. Lopes, Marcos R. Costa, Rodrigo R. Resende, Thiago M. Cunha, Akiva Mintz, Alexander Birbrair

**Affiliations:** ^1^ Department of Pathology Federal University of Minas Gerais Belo Horizonte Brazil; ^2^ Department of Pharmacology Federal University of Minas Gerais Belo Horizonte Brazil; ^3^ Brain Institute Federal University of Rio Grande do Norte Natal Brazil; ^4^ Department of Biochemistry and Immunology Federal University of Minas Gerais Belo Horizonte Brazil; ^5^ Department of Pharmacology University of São Paulo Ribeirão Preto Brazil; ^6^ Department of Radiology Columbia University Medical Center New York NY USA

**Keywords:** genetic depletion, melanoma, sensory nerves, tumour microenvironment

## Abstract

The tumour mass is composed not only of heterogeneous neoplastic cells, but also a variety of other components that may affect cancer cells behaviour. The lack of detailed knowledge about all the constituents of the tumour microenvironment restricts the design of effective treatments. Nerves have been reported to contribute to the growth and maintenance of numerous tissues. The effects of sensory innervations on tumour growth remain unclear. Here, by using state‐of‐the‐art techniques, including Cre/loxP technologies, confocal microscopy, in vivo‐tracing and chemical denervation, we revealed the presence of sensory nerves infiltrating within the melanoma microenvironment, and affecting cancer progression. Strikingly, melanoma growth in vivo was accelerated following genetic ablation or chemical denervation of sensory nerves. In humans, a retrospective analysis of melanoma patients revealed that increased expression of genes related to sensory nerves in tumours was associated with better clinical outcomes. These findings suggest that sensory innervations counteract melanoma progression. The emerging knowledge from this research provides a novel target in the tumour microenvironment for therapeutic benefit in cancer patients.

## 1 INTRODUCTION

Melanoma exhibits one of the most aggressive behaviours among cancers.^1^ It affects millions of people in the world.^2^ Promising therapeutic strategies have been refined in recent years, nevertheless the 5‐year overall survival of patients with metastatic cutaneous melanoma remains between 5% and 19%.^3^, ^4^ The disease is commonly originated as a consequence of the stepwise agglomeration of genetic and epigenetic alterations in melanocytes; nonetheless the tumour microenvironment plays a dynamic role in regulating the subsequent tumour growth.^5^ Physiologically, the skin microenvironment in healthy people is a physical and chemical barrier that protects from tumorigenesis; nevertheless, cancer cells evoke numerous changes to transform the adjacent normal cells into pathological entities.^6^ The orchestration of such events implicates expansion, migration and contribution of various cell types.^7^ The cellular composition of the tumour microenvironment is heterogeneous and yet not fully uncovered. While some components of this microenvironment promote,^8^, ^9^, ^10^ others limit tumour progression.^11^, ^12^, ^13^ Given the complexity and plasticity of the cancer‐associated cells, further studies are necessary in order to develop more effective therapies.

The skin is densely innervated, and nerves have been reported to contribute to its growth, functionality and maintenance.^14^ It has been recently reported that the two branches of the autonomic nervous system regulate cancer progression in different organs by controlling cancer initiation, progression and metastasis.^15^, ^16^, ^17^ Encouragingly, clinical studies describe positive effects of drugs that interfere with the autonomic nervous system in melanoma patients.^18^ In addition to autonomic nerves, the skin is also innervated by sparsely distributed sensory fibers, most of which express the voltage‐gated sodium (Nav) channel Nav1.8.^19^ The Nav1.8 channel can be specifically used as a molecular marker for sensory nerves, and has been targeted in research as a way to manipulate these nerves and study them within the skin as well as other tissues.^20^, ^21^, ^22^ Interestingly, in vitro co‐culture systems using sensory neurons from the dorsal root ganglion have suggested that nerve interactions may affect cancer cell proliferation.^23^ Sensory nerves can eventually contribute to tumour‐associated pain^24^, ^25^; yet, whether sensory nerve fibers are involved in tumour progression in vivo is unclear. Here, we have detected the presence of sensory innervations in the melanoma microenvironment and have tested the hypothesis that sensory nerves affect melanoma behaviour by evaluating the effect of genetic and pharmacologic ablations of these nerves.

## 2 MATERIALS AND METHODS

### 2.1 Animals

C57BL/6J wild‐type (WT) mice were obtained from the Central Animal Facility of the Federal University of Minas Gerais. *B6.TdTomato* (Stock number: 007914) and *B6.DTA* (Stock number: 006331) mice were purchased from Jackson Laboratories. *Nav1.8‐Cre* mice^26^ were obtained from Infrafrontier (EMMA ID: 04 582). *Nav1.8‐Cre* mice were bred with *B6.TdTomato* mice to generate Nav1.8‐Cre/TdTomato mice. Also, *Nav1.8‐Cre* mice were bred with *B6.DTA* mice to generate Nav1.8‐Cre^+^/DTA^+^ and control littermates (Nav1.8‐Cre^−^/DTA^+^).^27^ Age‐matched 8‐ to 10‐week‐old mice were used for experiments. All colonies were housed in a pathogen‐free facility of the Animal Research Program at of the Federal University of Minas Gerais under controlled light cycle (12:12‐hours light/dark cycle) and fed ad libitum. The Federal University of Minas Gerais Animal Care and Use Committee (CEUA) approved handling and procedures.

### 2.2 Cell culture

B16F10 mouse melanoma cells were obtained from the American Type Cell Culture (ATCC). Cells were cultured in RPMI (Sigma, San Louis, MO) + 10% Fetal Bovine Serum (FBS, Life Technologies, Carlsbad, CA) and used for in vivo experiments until the 5th passage.

### 2.3 In vivo analyses of tumour growth

For tumour growth, 8‐ to 10‐week‐old mice were injected with 5 × 10^4^ B16F10 cells subcutaneously in the right flank. Tumours were removed 16 days after injection and weighted. Length (L) and width (W) were measured for calculating tumour volume (V) using the formula V = 0.5 × (L × W^2^).^28^ Tumour area was determined using calibrated photographs of each tumour using Fiji software^®^, version 1.53 (National Institute of Health, Bethesda, MD).

### 2.4 RTX treatment

For chemical ablation of sensory nerves, WT mice were treated with resiniferatoxin (RTX, Sigma) as previously described.^27^ To perform ablation of sensory nerves before tumour implantation, 4‐week‐old mice were injected subcutaneously on consecutive days with increasing doses of RTX (30, 70 and 100 μg/kg) dissolved in 2% DMSO with 0.15% Tween 80 in PBS. Mice rested for 20 days before injection of B16F10 cells. For depletion of sensory nerves after tumour implantation, mice were injected subcutaneously with B16F10 cells and, after 48 hours, underwent treatment with increasing doses of RTX (30, 70 and 100 μg/kg). For both conditions, control mice were injected with vehicle alone. To access sensory depletion efficiency, mice were subjected to a behavioural test to measure the sensitivity to capsaicin, confirming the ablation of sensory nerves, as previously described.^29^ After intra‐plantar injection of capsaicin (3 μg in 20 μL), total licking time was recorded in a 5‐minute interval.

### 2.5 Immunohistochemistry and microscopy

After dissection, B16F10 tumours and dorsal root ganglions (DRG) were fixed overnight at 4°C in 4% buffered paraformaldehyde (PFA, pH = 7.4), incubated overnight at 4°C with 30% sucrose diluted in PBS, embedded and frozen in optimal cutting temperature compound (OCT, Tissue‐Tek). Embedded tissues were stored at −80°C. 20 μm cryosections were cut, and blocked for 2 hours in 3% BSA in PBS + 0.5% Triton and immunostained with the following antibodies: CD31‐PE (dilution 1:100) (BioLegend), Ki67 (dilution 1:100) (BD Biosciences), Guinea pig‐anti‐mouse‐Nav1.8 (dilution 1:500) (Merck Millipore), and anti‐Guinea pig‐AlexaFluor‐647 (1:1000) (Life Technologies). Sections were stained with DAPI and mounted in Dako fluorescence mounting medium (Dako, Santa Clara, CA). Stained sections were imaged on an inverted Zeiss LSM 880 confocal microscope (Oberkochen, Germany). CD31 area, vessel diameter and length, number of Ki67^+^ cells and Nav1.8 mean fluorescence intensity (MFI) were quantified using Fiji software^®^, version 1.53 (National Institute of Health). Multiple random fields of each section were used for quantification.

### 2.6 TUNEL assay

For analysing DNA fragmentation, tumour fragments were embedded in paraffin and 5 μm sections were used for TUNEL staining according to the manufacturer's protocol.^30^ Sections were deparaffinized, rehydrated and maintained in PBS. Permeabilization was performed for 20 minutes using Proteinase K (0.2 mg/mL in TBS). Endogenous peroxidases were inactivated using H_2_O_2_ (3% diluted in methanol). Sections were then labelled with terminal deoxynucleotidyl transferase (TdT) for one and a half hour and the reaction was terminated. Detection was performed using the conjugate and DAB provided by the manufacturer. Methyl green was used as counterstain and sections were analysed using a light microscope. Multiple fields of each section were analysed using Fiji software^®^, version 1.53 (National Institute of Health) to quantify the number of positive cells per area.

### 2.7 Kaplan‐Meier curves

Survival curves were plotted using the website R2: Genomics Analysis and Visualization Platform (http://r2.amc.nl). Genes commonly expressed by sensory nerves (Nav1.8, TRVP1 and VIP) were selected and the parameters chosen following the protocol given by the website.

### 2.8 Statistical analysis

Graphs were plotted using GraphPad Prism 7 (San Diego, CA). Shapiro‐Wilk normality test was performed, and unpaired *t* test was used to determine statistical significance.

## 3 RESULTS

### 3.1 Nav1.8+ sensory nerves infiltrate the tumour microenvrionment

**Figure 1:**
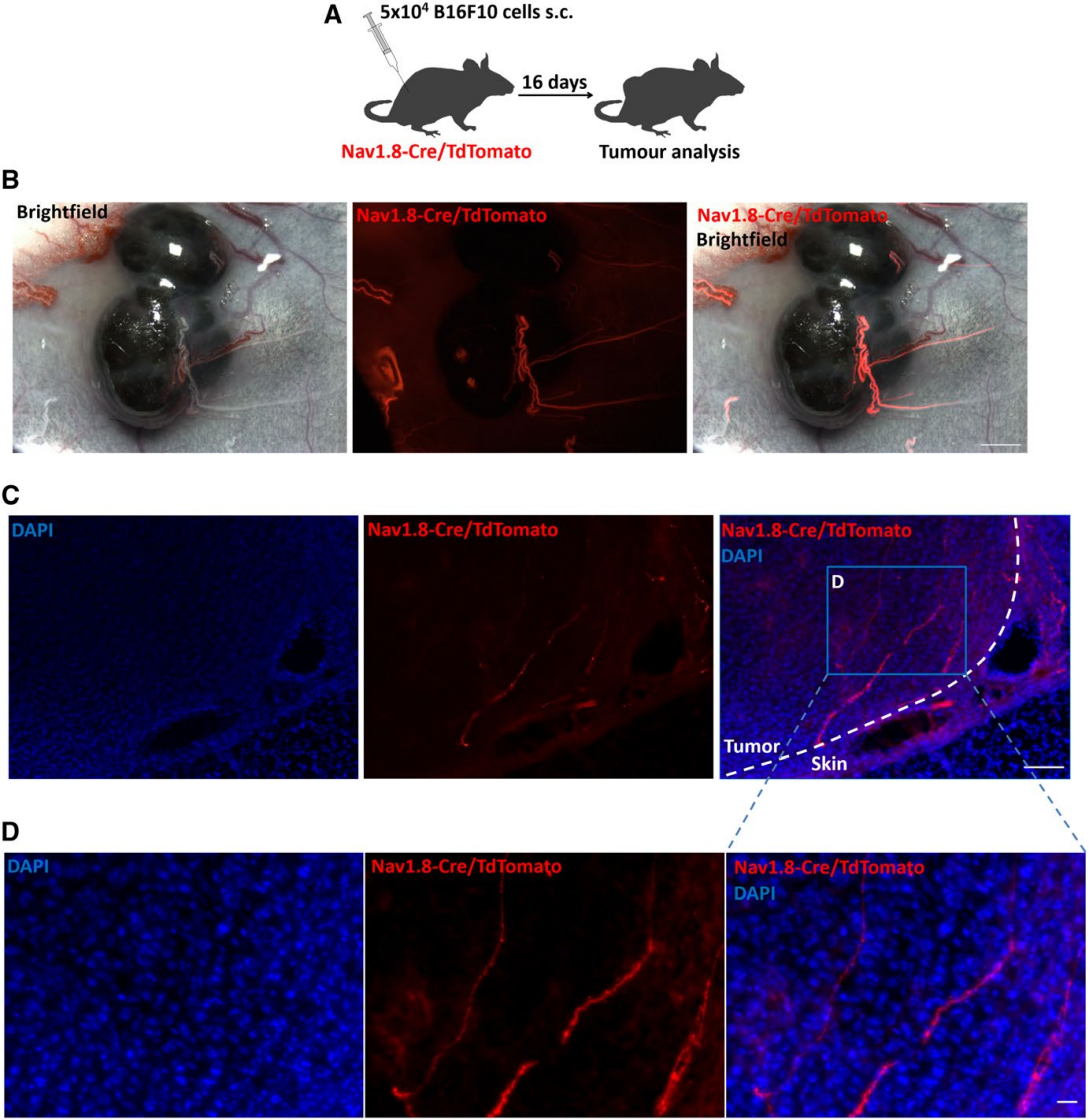
Sensory Nav1.8+ nerve fibers are present within the melanoma tumour microenvironment. A, Schematic representation for subcutaneous allograft melanoma growth. 5 × 10^4^ B16F10 melanoma cells were subcutaneously injected into Nav1.8‐Cre/TdTomato mice, and tumour tissues were surgically removed 16 days later. B, Whole melanoma tumour viewed from a fluorescent dissecting microscope showing Nav1.8^+^ sensory nerves labelled with TdTomato fluorescence (red) and brightfield images. C, Representative image of a Nav1.8‐Cre/TdTomato mouse tumoural section showing margin of the B16F10 melanoma. Dashed line indicates separation between tumour and normal adjacent skin. Nerve fibers are labelled with TdTomato fluorescence (red) and nuclei with DAPI (blue). TdTomato + sensory nerves are shown invading the tumour. D, High‐magnification image of C highlighting intra‐tumoural nerve fibers labelled with TdTomato fluorescence (red), nuclei are labelled with DAPI (blue). Scale bars: B = 1 cm; C = 100 μm; D = 20 μm

**Figure A1:**
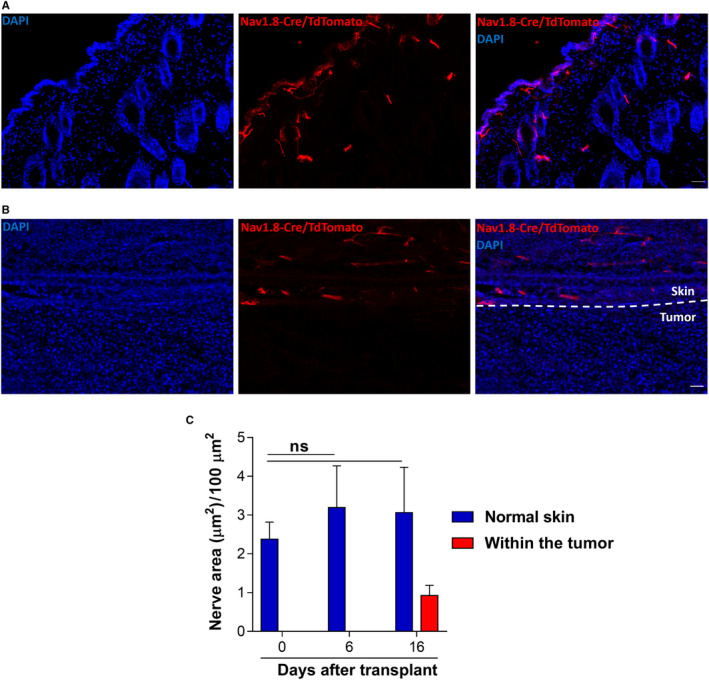
Sensory nerves dynamics in the adjacent normal skin and within the tumor during melanoma progression. A. Representative image of normal skin of Nav1.8‐Cre/TdTomato mice at the day of B16F10 cells transplantation. Identical skin areas are shown for different channels: DAPI (blue); Nav1.8‐Cre/TdTomato (red); and merged DAPI (blue) and Nav1.8‐Cre/TdTomato (red). B. Representative image of the skin of Nav1.8‐Cre/TdTomato mice 6 days after B16F10 cells transplantation. Dashed line separates tumor and the normal adjacent skin. Nerve fibers are labeled with TdTomato fluorescence (red) and nuclei with DAPI (blue). C. Quantification of sensory nerves area, at different time points during melanoma growth, in the normal adjacent skin (in μm^2^ per 100 μm^2^) (Day 0: 2.38 ± 0.44; day 6: 3.19 ± 1.07; day 16: 3.06 ± 1.17) and within the tumor (Day 16: 0.93 ± 0.26). Scale bars: A and B = 50 μm. Data are shown as mean ± SEM. Unpaired t test (ns p>0.05).

To examine whether sensory innervations are present within the tumour microenvironment, we have analysed an orthotopic melanoma tumour mouse model in which only sensory nerves are labelled with the red fluorophore TdTomato. We have crossed Nav1.8‐Cre mice with a mouse line conditionally expressing TdTomato.^21^ In Nav1.8‐Cre/TdTomato mice, upon removal of loxP‐stop‐loxP cassette by Cre recombination, TdTomato is expressed only in Nav1.8‐sensory neurons. We have transplanted B16F10 melanoma cells subcutaneously into Nav1.8‐Cre/ TdTomato immunocompetent C57BL/6 mice to analyse whether sensory innervations infiltrate the tumour during cancer progression. We have analysed sensory innervations in the area where B16F10 cells were transplanted immediately after injection and in tumours at 6 and 16 days after the transplantation. We observed no significant difference in sensory innervations present in the normal skin before the melanoma cells injection when compared to sensory innervations in the normal skin surrounding the tumours after 6 or 16 days (Figure A1A‐C). We did not detect sensory nerves within the tumours after 6 days (Figure A1B,C). Our analysis at 16 days post‐injection has revealed the presence of melanoma‐infiltrating sparsely distributed Nav1.8+ sensory nerve fibers, which were not observed at earlier time points (Figure 1A‐D; Figure A1A‐C). The length of these intra‐tumoural sensory nerves was 0.64 ± 0.18 µm per tumour area (mm^2^). Thus, these experiments demonstrated the presence of sensory nerves within the melanoma microenvironment. Nevertheless, whether they contribute to tumour progression remains unknown.

### 3.2 Genetic depletion of Nav1.8+ sensory nerves enhances melanoma growth and tumoural angiogenesis

**Figure 2:**
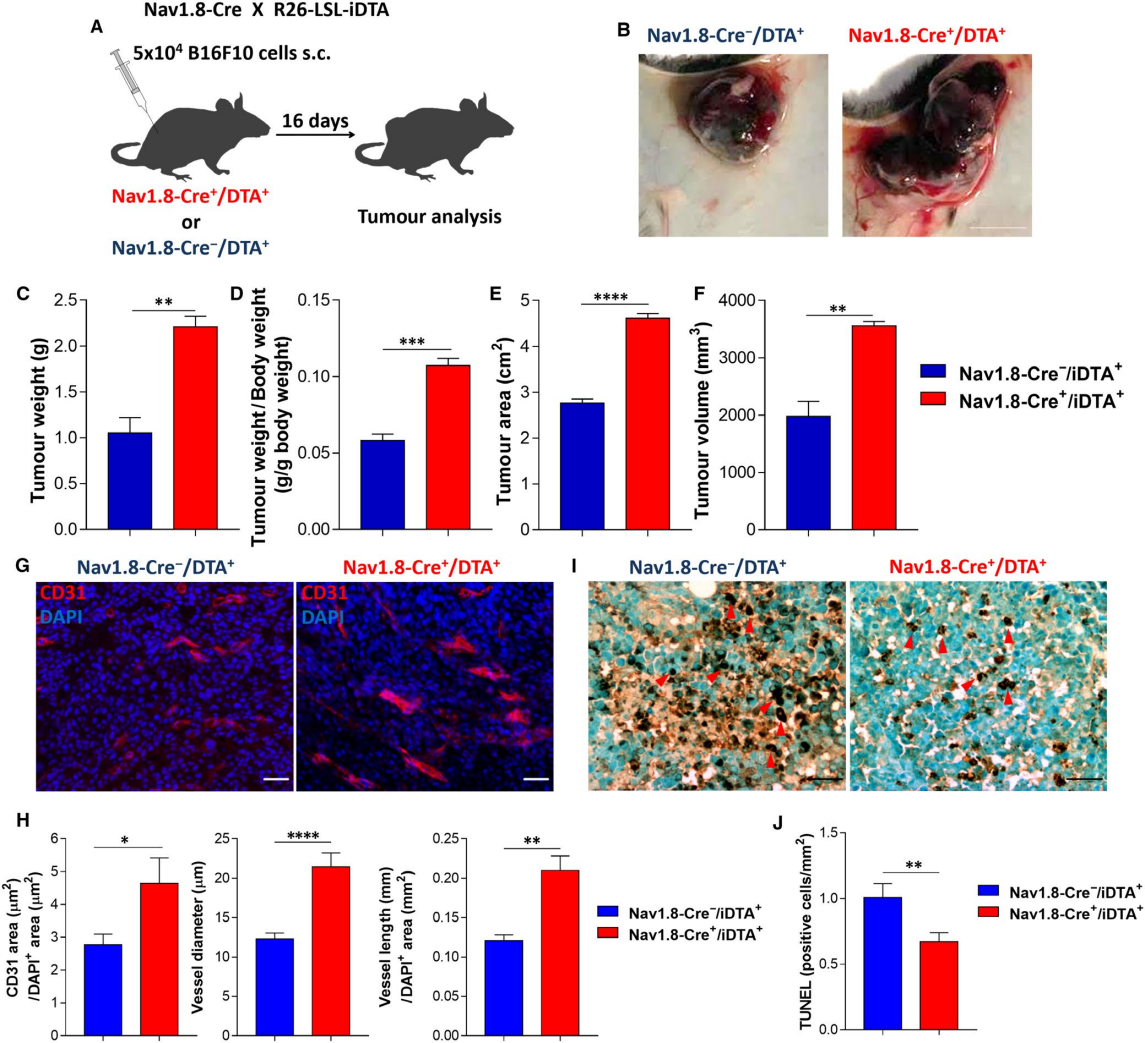
Genetic depletion of sensory Nav1.8^+^ nerve fibers enhances melanoma growth and increases intra‐tumoural angiogenesis. A, Schematic representation for subcutaneous allograft melanoma growth. 5 × 10^4^ B16F10 melanoma cells were subcutaneously injected into Nav1.8‐Cre^−^/iDTA^+^ (n = 5) and Nav1.8‐Cre^+^/iDTA^+^ (n = 5) mice, and tumours were removed for analysis after 16 days. B, Representative macroscopic image of B16F10 melanoma after dissection. C, Tumour weight (Nav1.8‐Cre^−^/iDTA^+^ 1.0 ± 0.2; Nav1.8‐Cre^+^/iDTA^+^ 2.2 ± 0.1). D, Tumour weight corrected by animal body weight (Nav1.8‐Cre^−^/iDTA^+^ 0.06 ± 0.004; Nav1.8‐Cre^+^/iDTA^+^ 0.11 ± 0.005). E, Tumour area (Nav1.8‐Cre^−^/iDTA^+^ 2.77 ± 0.09; Nav1.8‐Cre^+^/iDTA^+^ 4.61 ± 0.10). F, Tumour volume (Nav1.8‐Cre^−^/iDTA^+^ 1980 ± 264; Nav1.8‐Cre^+^/iDTA^+^ 3559 ± 76.5). G, Representative immunofluorescence images of tumours labelled for endothelial cells (CD31; red) to identify blood vessels and nuclei (DAPI; blue). H, Quantification of angiogenesis in melanomas by blood vessel area (Nav1.8‐Cre^−^/iDTA^+^ 2.8 ± 0.3; Nav1.8‐Cre^+^/iDTA^+^ 4.6 ± 0.8), diameter (Nav1.8‐Cre^−^/iDTA^+^ 12.3 ± 0.7; Nav1.8‐Cre^+^/iDTA^+^ 21.4 ± 1.7) and length (Nav1.8‐Cre^−^/iDTA^+^ 0.12 ± 0.01; Nav1.8‐Cre^+^/iDTA^+^ 0.21 ± 0.18). I, TUNEL immunohistochemical staining for DNA fragmentation of dead cells, red arrow heads indicate positive nuclei staining. J. Number of TUNEL‐positive cells per area (Nav1.8‐Cre^−^/iDTA^+^ 1.01 ± 0.11; Nav1.8‐Cre^+^/iDTA^+^ 0.67 ± 0.68). Scale bars: B = 1 cm; G and I = 50 μm. Data are shown as mean ± SEM. Unpaired *t* test (ns *P* > 0.05; * *P* < 0.05; ** *P*< 0.01; *** *P*< 0.001 and **** *P* < 0.0001)

**Figure A2:**
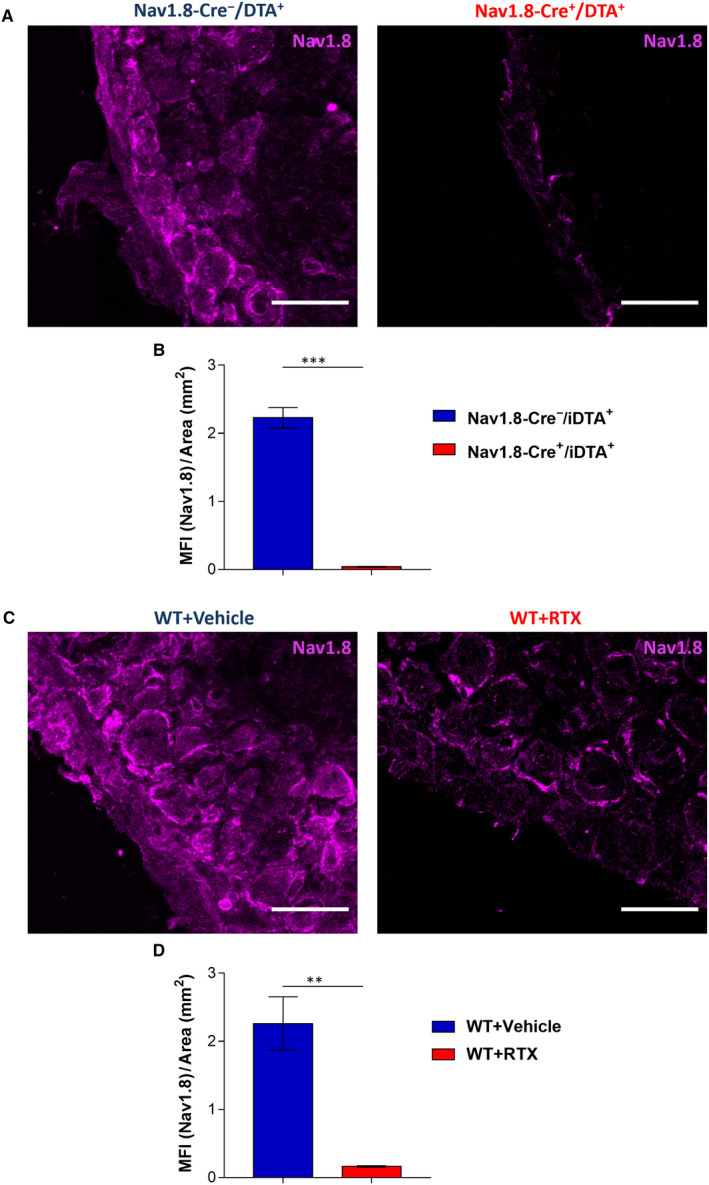
Nav1.8 expression decreases in the DRGs of Nav1.8‐Cre^+^/iDTA^+^ and RTX treated mice. A. Representative immunofluorescence images of DRGs labeled for voltage‐gated sodium channel 1.8 (Nav1.8; purple) from mice genetically depleted of sensory nerves Nav1.8‐Cre^+^/iDTA^+^ (n=5) and control littermates Nav1.8‐Cre^‐^/iDTA^+^ (n=5). B. Quantification of MFI per mm^2^ of DRG area from Nav1.8‐Cre^+^/iDTA^+^ (2.23 ± 0.15) and Nav1.8‐Cre^‐^/iDTA^+^ (0.04 ± 0.01) mice. C. Representative immunofluorescence images of DRGs labeled for voltage‐gated sodium channel 1.8 (Nav1.8; purple) from WT+RTX and WT+Vehicle mice. WT mice were injected daily with increasing doses of RTX (WT+RTX; n=6) or Vehicle (WT+Vehicle; n=8). Mice rested for 20 days before subcutaneous injection of 5x10^4^ B16F10 cells. DRGs were removed for analysis after 16 days. D. Quantification mean fluorescence intensity (MFI) per mm^2^ of DRG area from WT+Vehicle (2.26 ± 0.39) and WT+RTX (0.16 ± 0.01) mice. Scale bars: A and C = 60 μm. Data are shown as mean ± SEM. Unpaired t test (** p<0.002; *** and **** p<0.0001).

**Figure A3:**
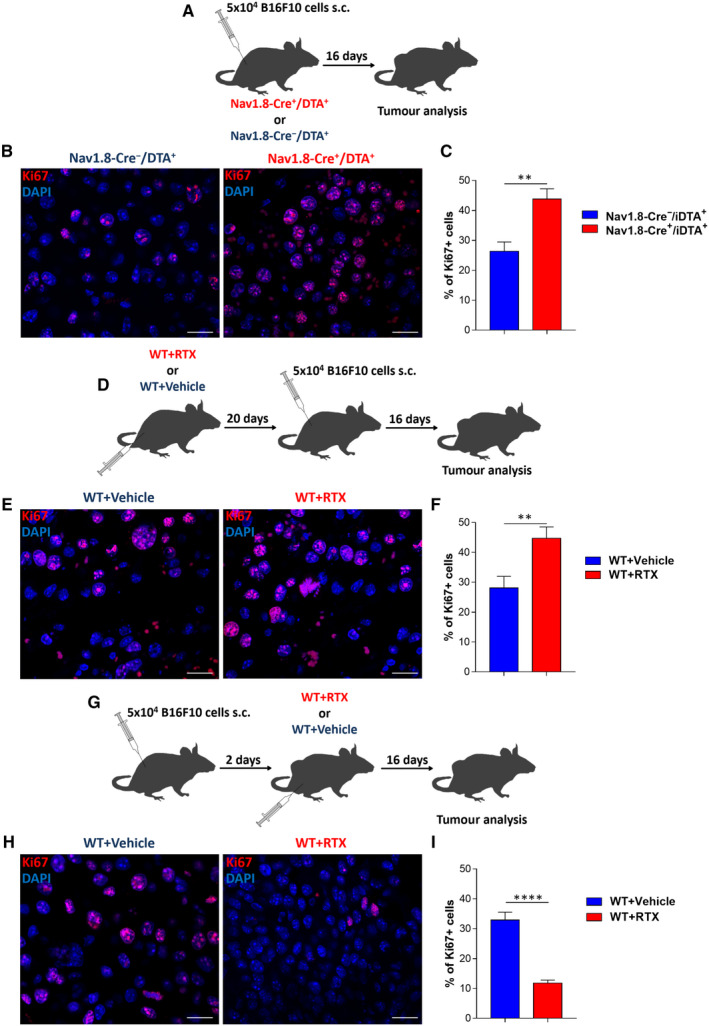
Proliferation within the tumors in response to sensory nerves ablation. A. Schematic representation for subcutaneous allograft melanoma growth in genetically denervated mice. 5x10^4^ B16F10 melanoma cells were subcutaneously injected into Nav1.8‐Cre^‐^/iDTA^+^ (n=5) and Nav1.80‐Cre^+^/iDTA^+^ (n=5) mice. Tumors were removed for analysis after 16 days. B. Representative immunofluorescent images of tumor sections labeled for proliferating cells (Ki67; red) and nuclei (DAPI; blue). C. Quantification of proliferating cells percentage within the tumors (Nav1.8‐Cre^‐^/iDTA^+^: 26.36 ± 3.10; Nav1.8‐Cre^‐^/iDTA^+^: 43.83 ± 3.39). D. Schematic representation for RTX‐mediated chemical depletion of sensory nerves followed by subcutaneous allograft melanoma growth. WT mice were injected daily with increasing doses of RTX (WT+RTX; n=6; 30, 70 and 100 μg/Kg) or vehicle (WT+Vehicle; n=8). Mice rested for 20 days before subcutaneous injection of 5x10^4^ B16F10 cells. Tumors were removed for analysis after 16 days. E. Representative immunofluorescent images of tumor sections labeled for proliferating cells (Ki67; red) and nuclei (DAPI; blue). F. Quantification of proliferating cells percentage within the tumors (WT+Vehicle: 28.12 ± 3.84; WT+RTX: 44.73 ± 3.81). G. Schematic representation for subcutaneous allograft melanoma growth followed by RTX‐mediated chemical depletion of sensory nerves. WT mice were injected at day 0 with 5x10^4^ B16F10 cells. RTX treatment (WT+RTX; n=3; 30, 70 and 100 μg/Kg) with increasing doses started 2 days after B16F10 cells injection. Control animals (WT+Vehicle; n=3) received vehicle instead. Tumors were removed for analysis after 16 days. H. Representative immunofluorescent images of tumor sections labeled for proliferating cells (Ki67; red) and nuclei (DAPI; blue). I. Quantification of proliferating cells percentage (WT+Vehicle: 32.93 ± 2.59; WT+RTX: 11.76 ± 1.02). Scale bars: B, E and H = 50 μm. Data are shown as mean ± SEM. Unpaired t test (** p<0.002; **** p<0.0001).

To explore the role of Nav1.8+ sensory nerves within the tumour microenvironment, we have induced targeted diphtheria toxin‐based cell ablation.^31^ We crossed Nav1.8‐Cre mice with inducible diphtheria toxin A (iDTA) transgenic mice to specifically deplete all sensory neurons.^26^ Nav1.8‐Cre/iDTA mice were previously shown to be devoid of all Nav1.8‐expressing nociceptors,^32^ and have no response to mechanical stimuli, noxious heat or capsaicin.^22^ Genetic depletion of sensory nerves was confirmed by immunohistochemistry to Nav1.8 in the dorsal root ganglions of these animals (Figure A2C,D). We have analysed the growth of B16F10 melanoma cells subcutaneously injected into Nav1.8‐Cre/ iDTA mice (genetically depleted of Nav1.8+ sensory nerves; Figure 2A). These experiments revealed that after 16 days, melanoma size increased in the absence of Nav1.8+ sensory nerves (tumour weight increased from 1.05 ± 0.17 to 2.21 ± 0.11 g; tumour weight per body weight increased from 0.06 ± 0.004 to 0.11 ± 0.005 g; tumour area increased from 2.77 ± 0.09 to 4.61 ± 0.10 cm^2^, tumour volume increased from 1980 ± 264 to 3559 ± 76 mm^3^; Figure 2B‐F). Also there was an enhancement in the intra‐tumoural blood vessels’ area (from 2.8 ± 0.3 to 4.6 ± 0.8 µm^2^), diameter (from 12.3 ± 0.7 to 21.4 ± 1.7 µm) and length (from 0.12 ± 0.01 to 0.21 ± 0.02 mm/µm^2^; Figure 2G,H). Additionally, tumour cells death decreased in the mice without sensory nerves (from 0.67 ± 0.7 to 1.01 ± 0.11 cells per mm^2^; Figure 2I,J). Genetic depletion of sensory nerves also led to an increase in proliferating cells within the tumour (from 26.36 ± 3.098 to 43.83 ± 3.394 per cent of cells within the tumour; Figure A3A‐C). Animal weights and blood counts were not affected by genetic ablation of sensory nerves in melanoma‐bearing mice (data not shown). Our results indicate that sensory nerves within the tumour microenvironment are trying to inhibit cancer progression.

### 3.3 Chemical depletion of sensory nerves before cancer cells implantation enhances melanoma growth and intra‐tumoural blood vessel formation

**Figure 3:**
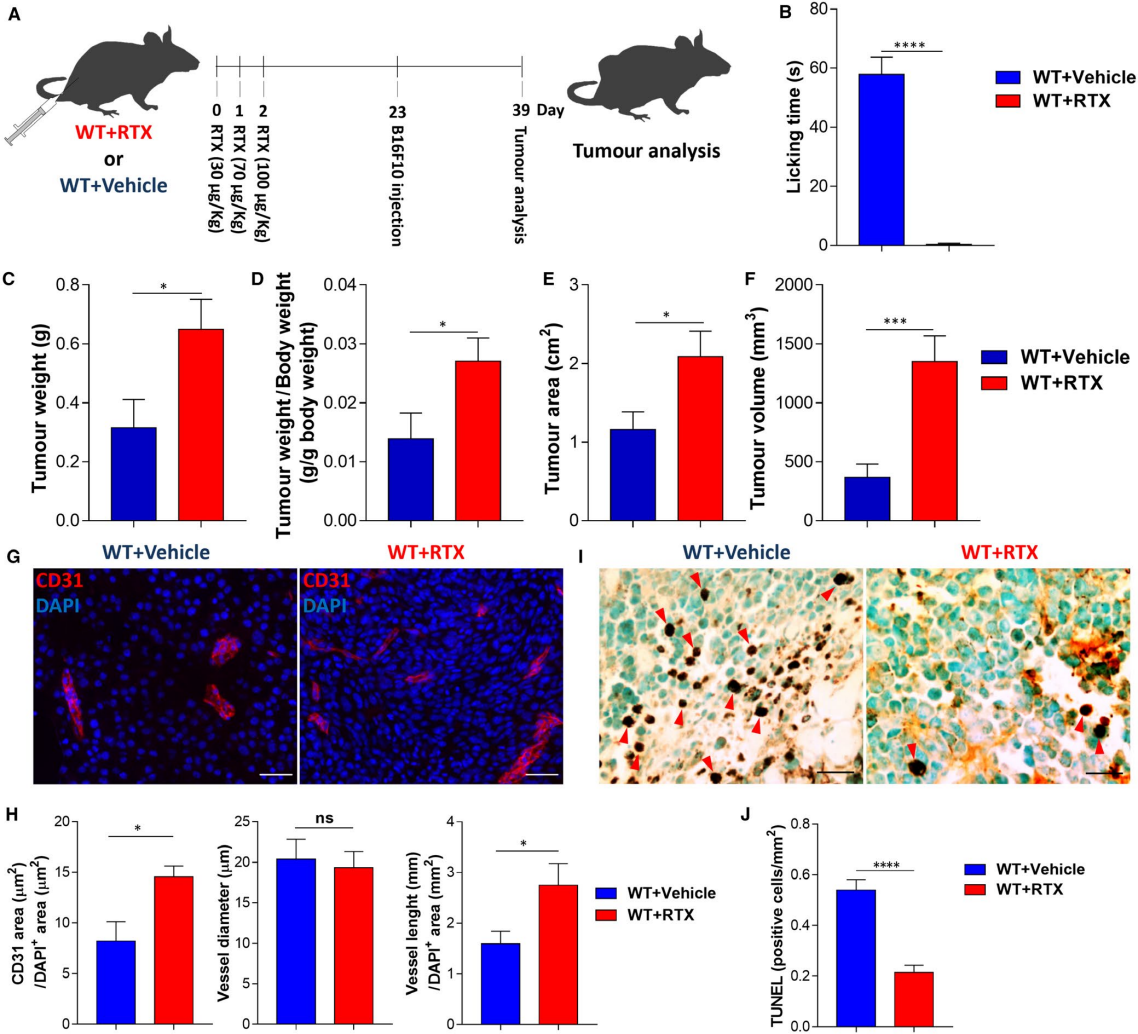
Chemical depletion of sensory nerves before melanoma cells transplantation enhances tumour growth and increases intra‐tumoural angiogenesis. A, Schematic representation for RTX‐mediated chemical depletion of sensory nerves and subcutaneous allograft melanoma growth. WT mice were injected daily with increasing doses of RTX (WT + RTX; n = 6; 30, 70 and 100 μg/kg) or vehicle (WT + Vehicle; n = 8). Mice rested for 20 days before subcutaneous injection of 5 × 10^4^ B16F10 cells. Tumours were removed for analysis after 16 days. B, Quantification of the time mice spent licking (Vehicle 57.9 ± 5.9; RTX 0.4 ± 0.4) the hind paw after injection of capsaicin (s.c. 3 μg per animal). C, Tumour weight (Vehicle 0.31 ± 0.09; RTX 0.65 ± 0.10). D, Tumour weight corrected by animal body weight (Vehicle 0.01 ± 0.004; RTX 0.03 ± 0.004). E, Tumour area (Vehicle 1.2 ± 0.2; RTX 2.1 ± 0.3). F, Tumour volume (Vehicle 366.6 ± 113; RTX 1350 ± 217). G, Representative immunofluorescence images of tumours labelled for endothelial cells (CD31; red) to identify blood vessels and nuclei (DAPI; blue). H, Quantification of angiogenesis in subcutaneously injected melanomas by blood vessel area (Vehicle 8.2 ± 1.9; RTX 14.5 ± 1.1), diameter (Vehicle 20.4 ± 2.4; RTX 19.3 ± 2) and length (Vehicle 1.6 ± 0.2; RTX 2.7 ± 0.4). I, TUNEL immunohistochemical staining for DNA fragmentation of dead cells, red arrow heads indicate positive nuclei staining. J, Number of TUNEL‐positive cells per area (Vehicle 0.54 ± 0.04; RTX 0.21 ± 0.03). Scale bars: 50 μm. Data are shown as mean ± SEM. Unpaired *t* test (ns *P* > 0.05; * *P* < 0.05; ** *P* < 0.01; *** *P* < 0.001 and **** *P*< 0.0001)

We sought an alternative method, for comparison, to confirm the role of sensory nerves in the melanoma microenvironment. Thus, we also achieved sensory denervation by treating wild‐type mice with resiniferatoxin (RTX), a capsaicin analogue.^33^ Pharmacologic depletion of sensory nerves was confirmed by immunohistochemistry to Nav1.8 in the dorsal root ganglions of these animals (Figure S2C,D). After pre‐treatment with 3 consecutive doses of RTX (30, 70 and 100 μg/kg), followed by melanoma cells transplantation, we analysed the tumour growth (Figure 3A,B). Pre‐treatment with RTX enhanced melanoma growth 16 days post‐transplantation of cancer cells (tumour weight increased from 0.31 ± 0.10 to 0.65 ± 0.10 g; tumour weight per body weight increased from 0.01 ± 0.004 to 0.03 ± 0.004; tumour area increased from 1.16 ± 0.22 to 2.09 ± 0.32 cm^2^, tumour volume increased from 367 ± 113 to 1350 ± 217 mm^3^; Figure 3C‐F). Additionally, there was an increase in the intra‐tumoural blood vessels’ area (from 8.2 ± 1.9 to 14.5 ± 1.1 µm^2^) and length (from 1.60 ± 0.25 to 2.75 ± 0.43 mm/µm^2^; Figure 3G,H). Moreover, tumour cells death decreased in RTX‐treated mice (from 0.54 ± 0.04 to 0.21 ± 0.03 cells per mm^2^; Figure 3I,J). Chemical depletion of sensory nerves before cancer cells transplantation also led to an increase in proliferating cells within the tumour (from 28.12 ± 3.84 to 44.73 ± 3.808 per cent of cells within the tumour; Figure A3D‐F). Animal weights and blood counts were not affected by sensory nerves depletion (data not shown). These results were similar to the ones achieved by genetic ablation of Nav1.8+ sensory nerves. Together, our results support the idea that sensory nerves try to block melanoma progression.

### 3.4 Resiniferatoxin (RTX) treatment after melanoma cells implantation reduces tumour growth

**Figure 4:**
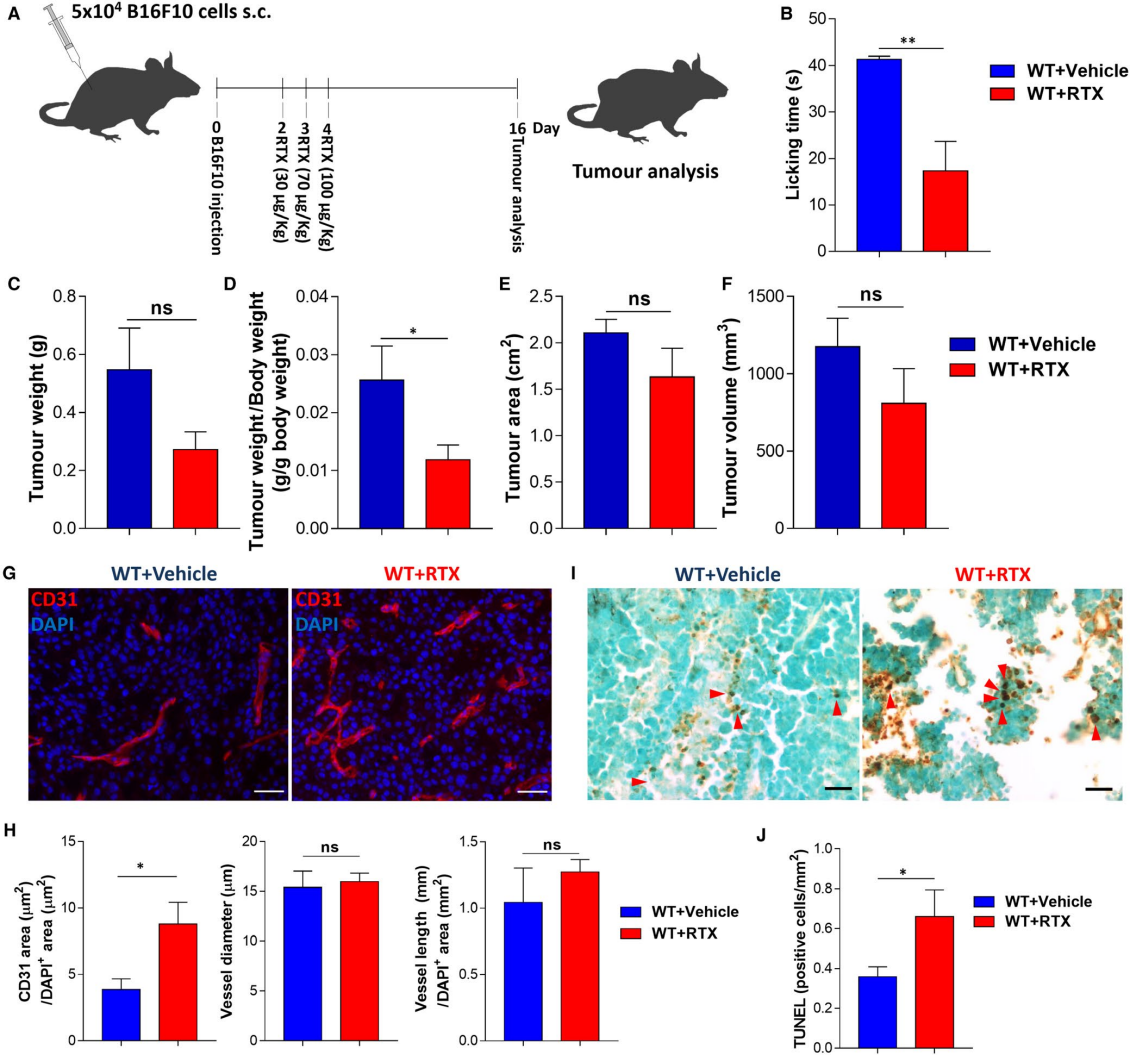
Resiniferatoxin (RTX) treatment after transplantation of melanoma cells reduces tumour growth. A, Schematic representation for subcutaneous transplantation of melanoma cells followed by RTX‐mediated chemical depletion of sensory nerves. WT mice were injected at day 0 with 5 × 10^4^ B16F10 cells. RTX treatment (WT + RTX; n = 5; 30, 70 and 100 μg/Kg) with increasing doses started 2 days after B16F10 cells injection. Control animals (WT + Vehicle; n = 3) received vehicle instead. Tumours were removed for analysis after 16 days. B, Quantification of the time mice spent licking (Vehicle 41.3 ± 0.7; RTX 17.3 ± 6.4) the hind paw after injection of capsaicin (s.c. 3 μg per animal). C, Tumour weight (Vehicle 0.55 ± 0.14; RTX 0.27 ± 0.06). D, Tumour weight corrected by animal body weight (Vehicle 0.03 ± 0.01; RTX 0.01 ± 0.002). E, Tumour area (Vehicle 2.11 ± 0.15; RTX 1.63 ± 0.31). F. Tumour volume (Vehicle 1175 ± 184; RTX 809 ± 225). G, Representative immunofluorescence images of tumours labelled for endothelial cells (CD31; red) to identify blood vessels and nuclei (DAPI; blue). H, Quantification of angiogenesis in subcutaneously injected melanomas by blood vessel area (Vehicle 3.9 ± 0.8; RTX 8.8 ± 1.6), diameter (Vehicle 15.4 ± 1.6; RTX 15.9 ± 0.9) and length (Vehicle 1.0 ± 0.26; RTX 1.3 ± 0.09). I, TUNEL immunohistochemical staining for DNA fragmentation of dead cells, red arrow heads indicate positive nuclei staining. J, Number of TUNEL‐positive cells per area (Vehicle 0.36 ± 0.05; RTX 0.66 ± 0.13). Scale bars: 50 μm. Data are shown as mean ± SEM. Unpaired *t* test (ns *P* > 0.05; * *P* < 0.05; ** *P* <0.01)

In contrast to our findings, some studies indicate a protective effect of capsaicin against several cancer types.^34^ Surprisingly, we discovered that the moment of RTX administration influences its effect on tumours' development. We analysed tumour growth in mice that were treated with 3 consecutive doses of RTX (30, 70 and 100 μg/kg) after subcutaneous transplantation of melanoma cells (Figure 4A,B). Interestingly, these mice presented reduced tumour size (tumour weight per body weight decreased from 0.03 ± 0.006 to 0.01 ± 0.002 g; tumour weight decreased from 0.55 ± 0.14 to 0.27 ± 0.06 g; tumour area decreased from 2.11 ± 0.15 to 1.63 ± 0.31 cm^2^, tumour volume decreased from 1175 ± 183.7 to 808.9 ± 224.7 mm^3^; Figure 4C‐F). Also, there was an increase in intra‐tumoural blood vessels’ area (from 3.9 ± 0.8 to 8.8 ± 1.6 µm^2^), while there was no difference in blood vessels’ diameter or length (Figure 4G,H). Additionally, tumour cells death increased in RTX‐treated mice (from 0.36 ± 0.05 to 0.66 ± 0.13 cells per mm^2^; Figure 4I,J). Chemical depletion of sensory nerves after cancer cells transplantation also led to a decrease in proliferating cells within the tumour (from 32.93 ± 2.589 to 11.76 ± 1.021 per cent of cells within the tumour; Figure A3G‐I). Animal weights and blood counts were not affected by RTX treatment after melanoma cells implantation (data not shown). This opposite effect of RTX given after cancer cells implantation, when compared to RTX pre‐treatment, is probably due to the direct effect of the drug on melanoma cells.^35^, ^36^

### 3.5 High expression of genes related to sensory nerves correlates with best outcomes in human cancer patients

**Figure 5:**
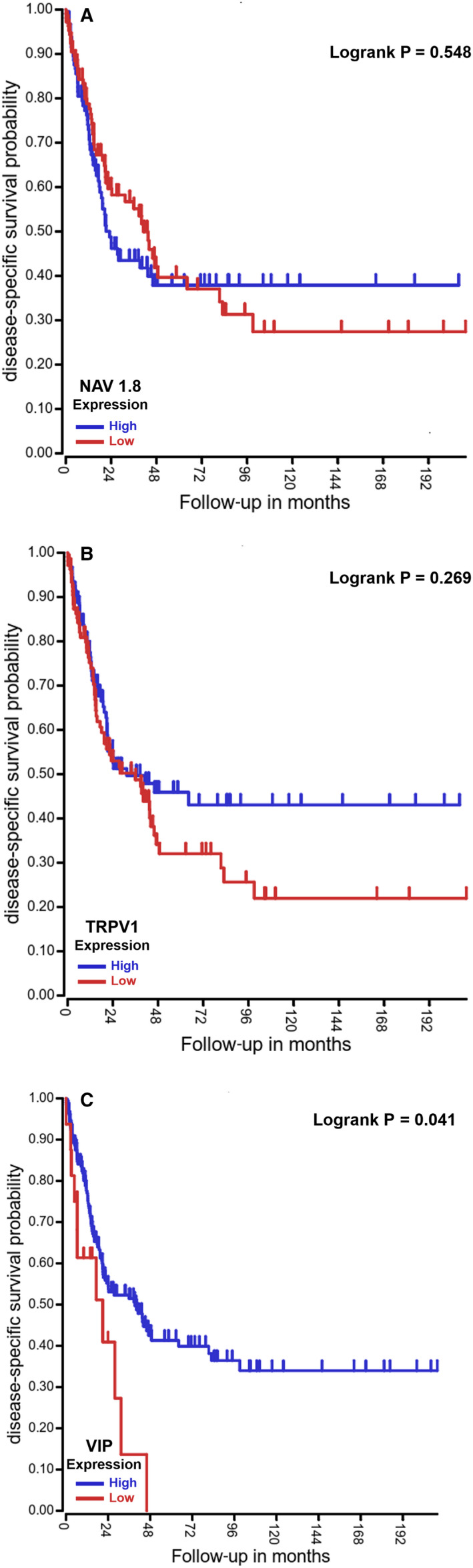
Low expression of sensory nerve‐related genes correlates with worse prognosis in melanoma. High expression of genes expressed in sensory nerves correlates with best outcomes in patients with melanoma. The prognostic impact of sensory nerve‐related genes in melanoma patients was evaluated using the R2: Genomics Analysis and Visualization Platform (http://r2.amc.nl). A, B, and C, We evaluated the survival probability of patients with melanoma based on their tumour transcriptome^38^ (n = 214). Kaplan‐Meier plots depicting the survival probability of patients with melanoma based on the expression of Nav1.8, TRPV1 and VIP in the tumours. A, High Nav1.8 expression is associated with improved outcome in patients with melanoma. B, High TRPV1 expression is associated with improved outcome in patients with melanoma. C, High VIP expression is associated with improved outcome in patients with melanoma. We queried a Kaplan‐Meier plotter data set.^38^ Log‐rank test was used

To better understand the role of sensory nerves in human cancer samples, we queried Kaplan‐Meier plotter data sets^37^ and evaluated the survival probability of patients with melanoma^37^ based on their tumour transcriptomes. In melanoma patients, the median transcript levels of genes expressed in sensory nerves [Nav1.8, TRPV1 and vasoactive intestinal peptide (VIP)] were used to stratify 214^37^ patient tumour transcriptomes into high and low expression for each gene. High expression of Nav1.8, TRPV1 and VIP was associated with a tendency to improved outcome in patients with melanoma (Figure 5A‐C). Future studies should analyse the expression of these genes in tumours from bigger groups of melanoma patients. These analyses are consistent with the data obtained in our mouse models: that the presence of sensory nerves in the tumour microenvironment was associated with suppressed melanoma growth. Although the expression of genes in tumour biopsies is used as a tool to characterize cancer subtypes and contribute to prognosis,^38^ during tumour progression some genes, including the ones related to sensory nerves, may be not specifically expressed by other cells within the tumours.^39^, ^40^ Therefore, these results should be also confirmed in future studies by immunohistochemical detection of sensory nerves within human melanoma biopsies correlated with prognosis.

## 4 DISCUSSION

**Figure 6:**
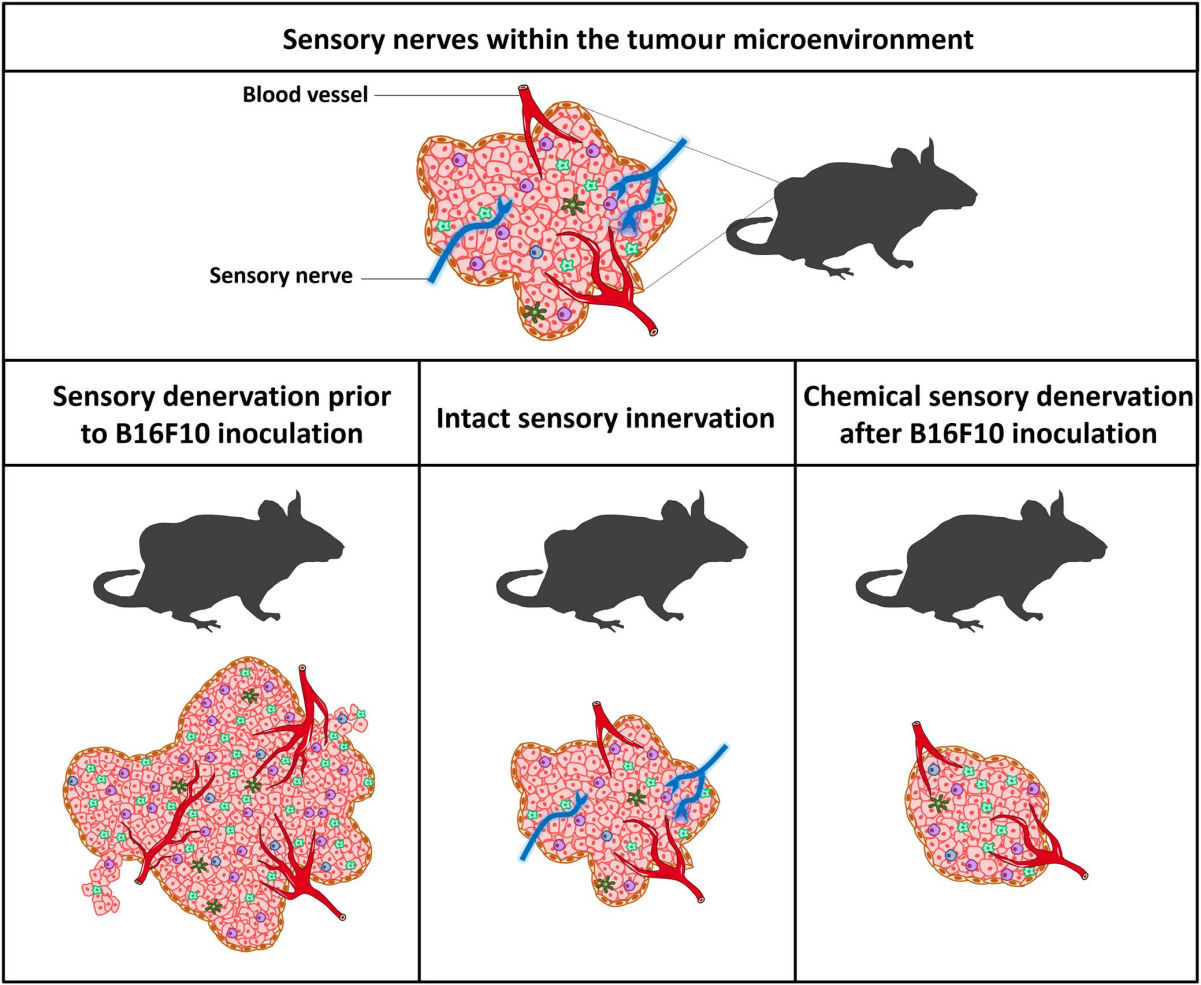
Schematic illustration summarizing the results of sensory nerves depletion from the tumour microenvironment. Sensory innervations, identified by their expression of Nav1.8, can be found in melanomas generated by B16F10 cell inoculation (Top). Genetic ablation of sensory Nav1.8+ nerve fibers increases tumour size. Similar results are obtained with chemical depletion of sensory nerves when resiniferatoxin (RTX) is given before tumour implantation (Bottom left). In contrast, when sensory nerves are chemically ablated after tumour implantation, tumour size decreases, probably due to the cytotoxic effect of RTX acting directly on tumour cells (Bottom right)

Here, we show the presence and importance of sensory nerves within the melanoma microenvironment. The sensory innervations are not inert within the tumours, but rather participate actively during tumour progression, as sensory nerve loss in a model of genetic depletion of Nav1.8+ sensory fibers or chemical depletion using RTX can induce changes in melanoma growth in vivo. Our results show that the sensory denervation leads to worse outcomes in melanoma‐bearing mice (Figure 6). Remarkably, we show that low expression of genes related to sensory nerves correlates with worse outcomes also in human melanoma patients, suggesting that sensory nerves try to block cancer growth.

**Figure 7:**
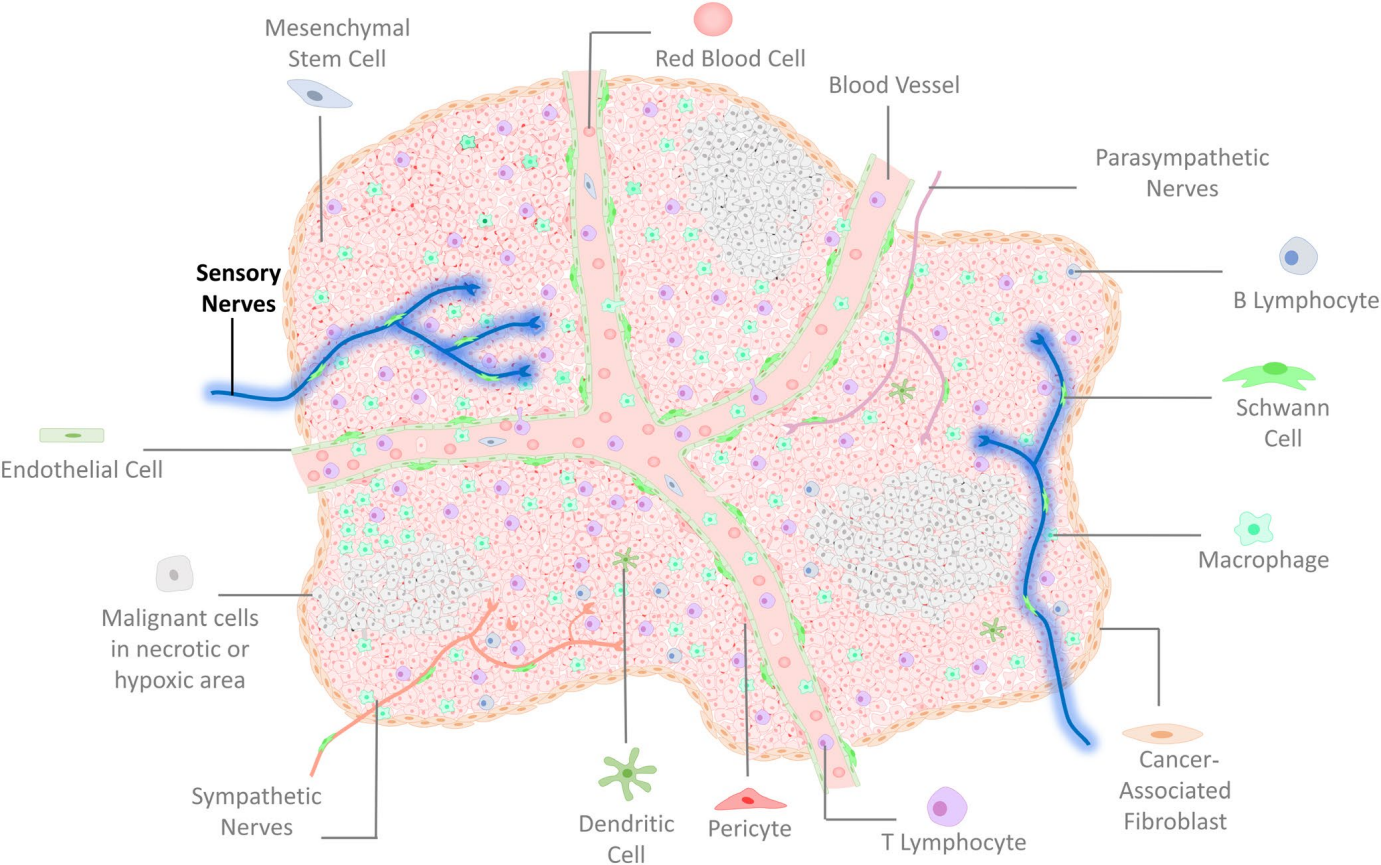
Schematic illustration showing the complexity of tumour microenvironment with its known components, outlining the newly discovered sensory nerves within the tumour

While several cellular and molecular mechanisms have been proposed to affect cancer progression, the identity and role of all tumour microenvironment components remain to be defined. The present results highlight a key constituent in the complex tumour microenvironment: sensory nerves (Figure 7). Previous studies have used chemical denervation of sensory nerves, by using capsaicin, nevertheless, given the possible broad unspecific effects of this drug, the question whether sensory nerves play a role in tumour progression remained open. Here, we proved by specific genetic ablation of sensory nerves that sensory innervation affects melanoma progression. Interestingly, our results are different from some recent studies that have shown capsaicin reducing cancer progression of tumour‐bearing mice, in models of pancreatic^41^ and prostate cancer.^42^ We show here that this is probably due to the moment of drug injection. As when we administered RTX after cancer cells implantation, our results also suggested that this drug inhibits tumour growth. Various previous studies have shown the direct effect of capsaicin on melanoma cells in vitro, inhibiting proliferation and migration, and increasing the death of these cells.^35^, ^43^, ^44^, ^45^, ^46^ Thus, when capsaicin, or its analog RTX, is administered after cancer cells implantation, it is already expected that tumour cells will be affected, and the role of sensory denervation cannot be analysed in this model without the interference of capsaicin’ direct effects on the tumour cells. On the other hand, sensory nerves’ genetic ablation or the treatment of mice with RTX before the cancer cells transplantation allowed us to analyse more specifically the role of sensory nerves in the tumour microenvironment in vivo.

Sensory nerves can contribute to tumour‐associated pain as demonstrated in pancreatic^24^ and prostate cancers.^25^ In vitro co‐culture systems using sensory neurons from the dorsal root ganglion have suggested that these nerves may interact with cancer cells.^23^ Interestingly, Keskinov and colleagues elegantly demonstrated that when co‐transplanted with dorsal root ganglion cultures, melanoma cells grow faster in vivo.^47^ Importantly, dorsal root ganglions are composed of a variety of components in addition to sensory nerves^48^; and the dorsal root ganglion culture may contain some non‐neuronal cells.^49^ Therefore, it is possible that melanoma growth in response to co‐transplantation with dorsal root ganglion cultures is influenced by other cells present within the dorsal root ganglion microenvironment. Thus, other studies are needed to dissociate the effects from transplanted sensory nerves and other non‐neuronal cells on melanoma growth in vivo. Importantly, dorsal root ganglion cultures did not up‐regulate proliferation of melanoma cells in vitro, raising the possibility that tumour growth comes from an indirect effect,^47^ or from a different source. Notably, it cannot be discounted that transplantation studies may include modifications in the neuronal properties by their preparation and grafting, possibly influencing their behaviour in vivo. For instance, the obtainment of dorsal root ganglion cultures demands axotomy of neuronal processes, which changes sensory nerves’ characteristics resembling the features of regenerating sensory innervations in vivo.^49^, ^50^ Consequently, the physiological role of endogenous sensory nerve fibers during melanoma progression remained unclear. Here, we show, by genetic and pharmacologic efficient ablation of endogenous sensory nerves (Figure S3A‐D), that sensory nerves are essential in the tumour microenvironment, and exert a protective effect against melanoma growth.

The present findings add to our understanding of the complexity in the pathogenesis of melanoma development. The detailed biological effects that result from genetic deletion of endogenous sensory nerves and from dorsal root ganglion transplantation remain to be explored further. Studying the effects of the loss of sensory nerves in geriatric microenvironment would also be interesting, as cancer incidence increases with age. There will be a need also to resolve the issue of whether there are subtypes of sensory neurons acting differently within the tumour microenvironment. Ultimately, resolution of the exact composition and nature of the sensory innervations probably awaits techniques that will allow single cell‐based analyses within the tumours.

It is likely that the molecular and cellular mechanisms involved in sensory nerves action are complex and may involve multiple pathways emerging from the tumour microenvironment that remain to be defined. It remains to be elucidated whether sensory nerves act directly, indirectly or both on cancer cells. Moreover, inflammation affects all stages of tumour development.^51^ Recent studies have shown that sensory neurons may activate inflammatory processes in several tissues.^21^, ^22^, ^52^ However, whether sensory nerves drive tumour inflammation was not analysed so far, and should be explored in future studies.

Developmental studies have provided evidence that ingrowth of sensory nerves precedes arterial blood vessel formation, which follows axons branching pattern in the embryonic skin.^53^, ^54^ However, it was not studied whether sensory nerves affect tumoural new blood vessel formation (angiogenesis). Our results indicate that sensory nerves inhibit tumoural angiogenesis within the melanoma. It remains unexplored how this happens, is this a direct or indirect effect of sensory nerves? Are sensory nerves affecting endothelial cells, pericytes or both? Sensory nerves endings can release neuropeptides, including substance P (SP), VIP, tachykinins, calcitonin gene‐related peptide (CGRP) and others.^55^ In this context, it will be interesting to discover which key signalling molecules are responsible for this phenotype in the tumour microenvironment.

Distinct peripheral nerves have been shown to be present in the tumour microenvironment of various organs, being implicated as regulators of cancer progression. Most studies suggest a pro‐tumorigenic neural role. The two branches of the autonomic nervous system have been shown to regulate prostate cancer progression: sympathetic adrenergic nerves are required for cancer initiation, while parasympathetic cholinergic fibers promote cancer metastasis.^15^, ^16^ Additionally, adrenergic and cholinergic signalling have been implicated in pancreatic^56^ and gastric^17^, ^57^ tumour progression, respectively. Whether our findings on sensory nerves apply to other organs remains unknown. Also, given the potential links within the peripheral nervous system, future research should evaluate whether different peripheral nerves within the tumour microenvironment communicate between them, and whether one innervation compensates for the absence of the other.

Histological proximity between malignant cells and peripheral innervations was described for many years exclusively as perineural invasion.^58^ In recent years, this concept has evolved, and new investigations are revealing that nerves may also play pro‐active roles within the tumour microenvironment, by regulating cancer progression.^17^, ^41^, ^56^, ^57^, ^59^, ^60^ Nevertheless, several questions still need to be answered. It remains to be elucidated whether functions of innervations are the same in distinct cancer types. Along the same lines, which cells in the tumour microenvironment are attracting the nerves? Are the attractants coming directly from cancer cells? For the progress of our knowledge on the roles of various innervations within the tumour microenvironment, more functional studies are needed. For instance, in vivo genetic conditional knockout experiments, by deleting key genes from specific intra‐tumoural nerves, will advance our understanding. These studies will clarify molecular mechanisms involved in the intra‐tumoural axonogenesis as well as in the interactions between nerves and other components present within the tumours.

A big challenge for the future will be to seek the translational value of these new findings to patients. Nerves may have promising clinical use not only as biomarkers, but also as therapeutic targets. Yet, more experimental investigations are needed to clarify the potential and discover specific targets within the tumour nerves for cancer management. The development of methods to manoeuvre intra‐tumoural innervations is yet at the earliest phases, and will demand the assembly of multidisciplinary groups to create nerves‐based treatments that can be used in the clinic. Among novel approaches, bioelectronic medicine may bring the possibility to control, by inhibition or stimulation, individual nerve fibers within the tumours, avoiding the off‐target side effects caused by most pharmacological drugs.^61^ Although enormous advancement has been achieved in targeting the tumour microenvironment to treat cancer, the best is yet to come.

In conclusion, our results suggest that protection of endogenous sensory nerves in cancer patients may thus provide an stimulating new avenue for anti‐cancer therapy. Taking into account our evidence regarding sensory nerves anti‐tumoural role, expanding the research into the mechanisms by which sensory innervations act blocking tumour growth may lead to the identification of potential new therapeutic pathways. This work raises the exciting and novel concept that targeting the peripheral sensory nervous system in the tumour might provide a novel approach to treat melanoma. Additionally, this study lays the groundwork for a paradigm that may have a broad impact on our understanding and the management of other cancers as well.

## CONFLICT OF INTEREST

The authors indicate no potential conflicts of interest.

## AUTHOR CONTRIBUTION

PHDMP, CL, WNS, AM and AB designed the study; PHDMP, CL, WNS, BGSR, GSPS, ACC, CCP, IFGS, WAG, MSV, PACC and LMCCC performed the experiments; PHDMP, CL, WNS, BGSR, GSPS, ACC, CCP, IFGS, WAG, MSV, PACC, LMCCC, RRR, TMC, AM and AB analysed data; MTP, MRC and TMC contributed reagents/analytical tools; AB supervised the study. PHDMP, CL, WNS, AM and AB interpreted data and wrote the manuscript. All authors discussed the results and commented on the manuscript.

## Data Availability

The data that support the findings of this study are available on request from the corresponding author.
